# Characteristics and Driving Mechanism of Water Resources Trend Change in Hanjiang River Basin

**DOI:** 10.3390/ijerph20043764

**Published:** 2023-02-20

**Authors:** Ming Kong, Jieni Zhao, Chuanfu Zang, Yiting Li, Jinglin Deng

**Affiliations:** School of Geography, South China Normal University, Guangzhou 510631, China

**Keywords:** water resources, SWAT model, spatial-temporal evolution, Mann-Kendall test, Hanjiang River Basin

## Abstract

Studying the historical and future trends of water resources in a basin and explaining the causes of water resource changes is very important, which is key to the management of water resources in a basin. The Hanjiang River Basin is an important water supply source for southwestern Fujian and eastern Guangdong, but it has an uneven spatial and temporal distribution of water resources and an outstanding conflict between supply and demand. In this study, the SWAT model was used to simulate the conditions of the Hanjiang River Basin in the last 50 years, using long time series climate data to study the characteristics and driving mechanism of water resources trend change. The results show that the water resources in the basin have not increased significantly in the last 50 years, but evapotranspiration has increased significantly. The forecast results for water resources in the future are reduced. The water resource changes in the basin have been unevenly distributed in the last 50 years. Climate change has been the main factor in total water resource change in the basin, while the difference in water resource change trends within the basin is caused by land use. The key reason for the decrease in water resources in the Hanjiang River Basin is the significant increase in evapotranspiration due to the significant increase in temperature. If this situation continues, the available water resources in the basin will continue to decline. In fact, many basins around the world are currently likely to have such problems, such as the 2022 summer drought in the Danube River Basin in Europe and the Yangtze River Basin in China, so this article is informative and representative of future water resources management in the basin.

## 1. Introduction

Water resources are one of the most important factors in maintaining natural equilibrium, ensuring human existence, and fostering socioeconomic growth. They are essential for human consumption, agricultural irrigation, energy, and industrial production, and support significant water-dependent activities [1]. Climate change has a big impact on how water resources change. Among the many climate factors, variations in precipitation and temperature have an enormous influence on the hydrological response cycle [2,3]. According to the Intergovernmental Panel on Climate Change (IPCC), the effects of climate change have led to the current patterns in hydro-meteorological time series, particularly over the past 30 years [4]. Understanding the evolution of water resources requires analyzing how climate change affects water resources and how the water cycle reacts to environmental changes and their causes. The study of the characteristics of water resources in a basin can characterize the seasonal and annual changes of natural processes, reveal the spatial evolution characteristics of water resources in the basin over time, and reveal the most direct way of revealing the past changes of a certain element [5]. In the context of climate change and the global water crisis, studying the spatial and temporal patterns and trends of water resources in a basin from the perspective of long-term sequences and using mathematical models to predict future trends in water resources are very important for understanding water resource changes and developing scientific management systems.

When studying water resource changes, it is important to consider how much, how, and why they occurred. To illustrate the severity of water stress and expected trends in several Mediterranean Sea locales, Milano et al. studied the current condition of water resources in the Mediterranean Sea and projected developments under anthropogenic and climate change [6]. Christensen et al. compared the U.S. Department of Energy/National Center for Atmospheric Research Parallel Climate Model (PCM) by comparing downscaled climate simulations derived from simulated hydrology and water resources scenarios to observed historical (1950–1999) climate-driven scenarios to assess the potential effects of climate change on hydrology and water resources in the Colorado River Basin [7]. It is evident from earlier research that the issue of water resource change in a basin may be better investigated by looking at the phenomena of water resource change in conjunction with elements such as climate change. Finding the fundamental mechanisms at work is also essential. Using information from two runoff stations and four rainfall stations in the South African watershed Limpopo River between 1949 and 2015, Nyikadzino et al. [8] calculated the monthly mean rainfall and runoff. Sen’s Slope and Mann-Kendall tests were carried out to determine trends and the station’s statistical significance for precipitation and runoff. Current trend variability studies typically concentrate on a single meteorological variable or hydrological indicator and analyze interannual variability or seasonal characteristics on a time scale [9,10]. Such trends can be used to investigate the spatial and temporal evolution patterns of water resources in a watershed if they can be spatially depicted. It can show how water resources have changed. Setting up a scientific system for managing water resources and increasing the effectiveness of their utilization requires the spatial-temporal evolution pattern of climate [11].

Field research in watershed hydrology continues to recognize and document the enormous diversity and complexity of rainfall-runoff processes as more and more watersheds, hydroclimatic regimes, and scales are considered [12]. Accurate modeling of watersheds and the interpretation of hydrological responses are crucial due to the complexity of watershed responses to spatially and temporally variable climate inputs and global changes [13]. It is anticipated that lessons learned from watershed-scale studies can be applied to global hydrological studies [12,14]. The Hanjiang River is prominent in eastern Guangdong and southwestern Fujian; it serves as a vital water source for all basin regions and a substantial outflow water source. Although there are often enough water resources in the basin, they are not distributed equally throughout time and area. The dichotomy between water availability and demand is getting worse, and it does not fit the pattern of regional productivity. At present, there are few academic studies on the Hanjiang River Basin, but the planning of water resources in the Hanjiang River Basin is an important issue for the sustainable development of the Guangdong-Hong Kong-Macao Greater Bay Area and its surrounding areas in Guangdong Province, China. Therefore, this study takes the water resources of the Hanjiang River Basin as the research object and studies the spatial and temporal distribution characteristics of water resources in the dry and wet seasons, as well as the historical and future variation characteristics of water resources over a long time series. Data from the SWAT model’s simulations of precipitation, temperature, and water resources during the basin’s 50 years served as the basis for the analysis. This article aims to provide statistical support and a scientific framework for managing water resources in the Hanjiang River Basin by projecting the future trend. It also complements expertise in watershed hydrological modeling.

## 2. Materials and Methods

### 2.1. Study Area

The Hanjiang River Basin encompasses 22 cities and counties in the provinces of Guangdong, Fujian, and Jiangxi and is located between 115°13′117°09′ E and 23°17′26°05′ N. The meeting of the Mei River and the Ting River creates the upstream portion of the Hanjiang River Basin. The Mei River rises in Shangfeng, Zijin County, Guangdong Province. It runs from southwest to northeast through the towns and counties of Wuhua, Xingning, Meixian, Meizhou, and Tai Po in Guangdong Province. The Ting River rises in Laijiashan, Ninghua County, Fujian Province, and flows from north to south through Changting, Wuping, Shanghang, and Yongding. It is named Hanjiang and is located near the Mei and Ting rivers near Sanhe Dam in Tai Po County. The Hanjiang River Basin area is 30,112 km^2^, and the mainstream length is 470 km, The annual runoff modulus is 26.7 dm^3^s^−1^km^2^, and the current annual runoff is 24.5 billion m^3^. 600–1200 mm is the typical depth of multi-year runoff. Throughout the year, runoff is distributed differently. The highest flood flow ever recorded at Chaoan station is 13,300 m^3^/s.

**Figure 1:**
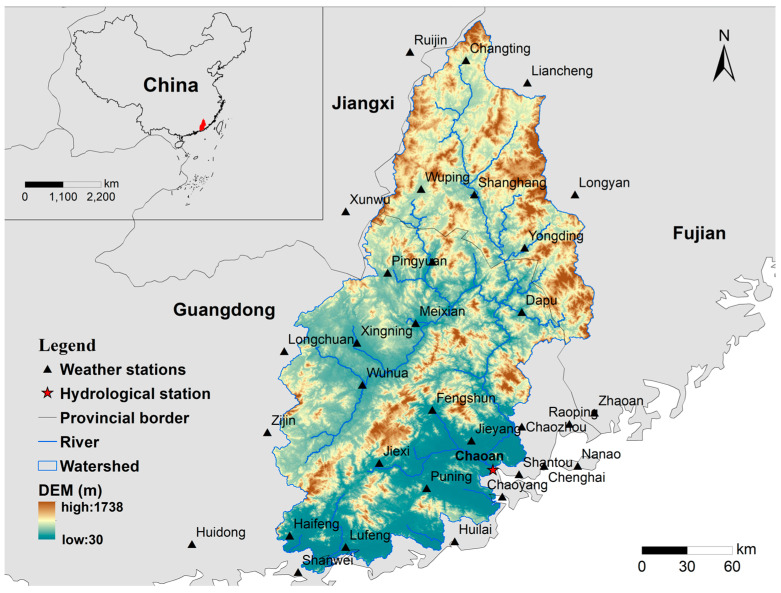
Location and distribution of meteorological and hydrological stations in Hanjiang River Basin, China.

The subtropical Southeast Asian monsoon region, where the Hanjiang River Basin is located, has a subtropical climate and is heavily influenced by the oceanic Southeast Asian monsoon. This region experiences year-round high temperatures and humidity and an abundance of rainfall, with an average of 1450–2000 mm annually. In the Hanjiang River Basin, precipitation is unevenly distributed throughout the year. April to September is the wet season, with ample precipitation accounting for around 80% of the year. From April to June, the rain is mostly frontal; from July to September, it is mostly typhoon rain. The basin normally experiences greater floods from April to September, most of which are brought on by typhoon rain. January through March and October through December are the dry seasons, and 15% to 30% of the year is made up of precipitation. Therefore, the average discharge is 2.7 times greater during the flood season than during the dry season [15]. The uneven rainfall distribution throughout the year is to blame for the disparity in water resources between the dry and wet seasons in the Hanjiang River Basin [16] (Figure 1).

### 2.2. Materials

The Hanjiang River Basin provided the elevation data (DEM), weather data, land use data, and soil data used in this study. The above data is collected, preprocessed, and imported into the basin to create a distributed hydrological model (SWAT). The specific information and sources are listed in Table 1.

### 2.3. Soil and Water Assessment Tool (SWAT)

The Soil and Water Assessment Tool (SWAT) model is based on physical principles, is deterministic and continuous, and is used in watershed-scale simulations [11,17]. The model is one of the most widely used models in the world, and its design modules are comprehensive and advanced enough to meet client requirements. For the time being, SWAT models are primarily used in three areas: hydrological studies and the evaluation of water resources [18]; the assessment of water quality, including non-point source pollution, sediment loss, and agricultural best management practices (BMPs) [19]; and the analysis of changes in land use and land management as well as the impacts of climate change. This model has been used in several experimental studies to assess and simulate the effects of climate change and land use changes [20]. The SWAT model was used in this study to simulate the history of water resources more correctly in the Hanjiang River Basin and to investigate the effects of changing environmental variables on hydrological conditions.

Using land use data, meteorological data collected from 1970 to 2020, and soil type data, ArcSWAT was utilized to run monthly and annual scale runoff simulations for the Hanjiang River Basin. The hydrological cycle follows the water balance equation given below:

(1) $${SW}_{t}={SW}_{0}+\sum_{i=1}^{t}\left(P_{day}-Q_{surf}-E_{a}-W_{seep}-Q_{gw}\right)$$

In Equation (1), t is the time step (d), $P_{day}$ is precipitation (mm), $Q_{surf}$ is surface runoff (mm), $E_{a}$ is evapotranspiration (mm), $W_{seep}$ is soil infiltration and lateral flow (mm), and $Q_{gw}$ is subsurface runoff. ${SW}_{t}$ and ${SW}_{0}$ are the final and starting soil water contents, respectively (mm).

### 2.4. Model Calibration and Validation

The SUFI2 algorithm is a method for calculating the objective function by randomly generating a set of parameters substituted into SWAT by the Latin-Hypercube simulations. In this study, the calibration and uncertainty procedure SWAT-CUP, combined with the SUFI2 algorithm, was applied to handle the sensitivity analysis and parameter rate setting of the model. The model was calibrated and validated using runoff data from Chaoan County hydrological station from 1980 to 2010, with 1981 to 1995 as the rate period and 1996 to 2010 as the validation period. The correlation coefficient $R^{2}$ and Nash coefficient $E_{ns}$ between the observed and simulated values were used to determine the applicability of the SWAT model in the Hanjiang River Basin [21]. The formulas for calculating $R^{2}$ and $E_{ns}$ are as follows.

(2) $$\begin{array}{c} R^{2}=\frac{{\left[\sum_{i=1}^{n}\left(Q_{Oi}-\bar{Q_{O}}\right)\left(Q_{mi}-\bar{Q_{m}}\right)\right]}^{2}}{\sum_{i=1}^{n}{\left(Q_{Oi}-\bar{Q_{O}}\right)}^{2}\sum_{i=1}^{n}{\left(Q_{mi}-\bar{Q_{m}}\right)}^{2}} \end{array}$$

(3) $$\begin{array}{c} E_{ns}=1-\frac{{\sum_{i=1}^{n}\left(Q_{mi}-Q_{Oi}\right)}^{2}}{\sum_{i=1}^{n}\left(Q_{Oi}-\bar{Q_{O}}\right)} \end{array}$$

In Equations (2) and (3), $Q_{Oi}$ and $Q_{mi}$ are the measured and simulated values, respectively; $\bar{Q_{o}}$ is the mean value of measured data, $\bar{Q_{m}}$ is the mean value of simulated data, and n is the number of data points.

**Figure 2:**
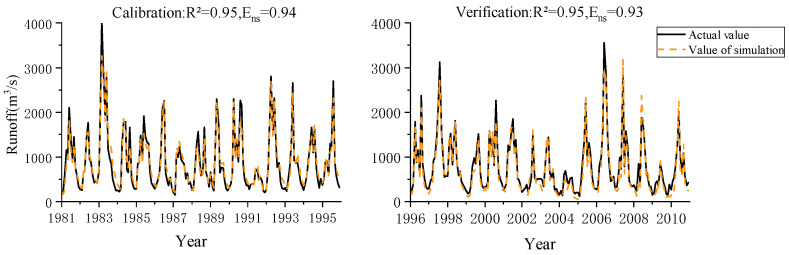
Simulated runoff and measured runoff for model rate determination and validation.

The model’s calibration results were R²=0.95 and $E_{ns}=0.94$, and the validation results were R²=0.95 and $E_{ns}=0.93$ (Figure 2). The higher the coefficient $R^{2}$ is, the closer the simulated runoff is to the measured runoff [11]. The high values of $R^{2}$ and $E_{ns}$ in this study indicate that the SWAT model of the Hanjiang River Basin is successfully constructed with high simulation accuracy, which can meet the requirements of the study.

### 2.5. Water Resource Calculation

The total lateral flow in the root zone, surface runoff, and seepage constitutes the water resources [11,22]:

(4) $$W=\sum_{i=1}^{n}\left(PERC+SURQ+LATQ\right)\times S_{i}\times 1000$$

In Equation (4), W denotes total water resources (m^3^); PERC denotes water seepage in the root zone (mm); SURQ denotes surface runoff; LATQ denotes lateral flow; $S_{i}$ denotes the catchment area of the $i^{th}$ HRU (km^2^); n denotes the number of hydrological response units.

### 2.6. Trend Analysis Methods

The Mann-Kendall (M-K) statistical test was used in this study to evaluate and test the trend of total water resources in the Hanjiang River Basin over the last 50 years (1970–2020). The S-M-K method was used to screen for abrupt changes in water resources and climate factors. The Sen’s Estimator (S-E) was used to investigate the magnitude of changes. The Hanjiang River Basin’s total water resources and precipitation temperature were forecast using the Hurst index, and the effects of five meteorological variables—precipitation, temperature, air pressure, humidity, and wind speed—on changes in water resources were investigated using multiple linear regression analysis.

#### 2.6.1. Mann-Kendall Non-Parametric Statistical Test

For time-series trend analysis of environmental data, the World Meteorological Organization recommends using the Mann-Kendall (M-K) non-parametric statistical test. Compared with other trend analysis techniques, it is free of outlier interference and samples that follow a specific distribution. It is frequently used to study trends in hydro-meteorological data, such as water quality, flow, temperature, and rainfall series. It has higher applicability for non-normal meteorological and hydrological data analysis [4]. In essence, non-parametric statistical methods compare the rank of the data series rather than the actual data values to determine the correlation between two variables. This eliminates the impact of extremely large and extremely small values on the outcomes of hydrological studies and enables a more objective assessment of whether the data series shows a changing trend [11]. The mathematical principle of the M-K test is as follows: assuming $x_{1},x_{2},\ldots x_{n}$ are time series variables, and n is the length of the time series. The M-K test defines the statistic S. S is calculated using the following equation:

(5) $$S=\sum_{k=1}^{n-1}\sum_{j=k+1}^{n}sgn\left(x_{j}-x_{k}\right)$$

In Equation (5), $x_{j}$ and $x_{k}$ are the observed values in year *j* and year *k*, j>k, n is the record length of the series, and $sgn\left(x_{j}-x_{k}\right)$ characterizes the following functions.

(6) $$sgn\left(x_{j}-x_{k}\right)=\left\{\begin{array}{c} 1,x_{j}-x_{k}>0\\ 0,x_{j}-x_{k}=0\\ 1,x_{j}-x_{k}<0 \end{array}\right.$$

the random sequence $S_{i}(i=1\ldots n)$ approximately obeys the normal distribution:

(7) $$Var\left(S\right)=\frac{1}{18}\left[n\left(n-1\right)\left(2n+5\right)-\sum_{t}t\left(t-1\right)\left(2t+5\right)\right]$$

the statistical test value Z is calculated using the following equation:

(8) $$Z=\left\{\begin{array}{c} \frac{S-1}{\sqrt{Var\left(S\right)}},S>0\\ 0,S=0\\ \frac{S+1}{\sqrt{Var\left(S\right)}},S<0 \end{array}\right.$$

In Equations (7) and (8), Z is a normally distributed statistic, and $Var\left(S\right)$ is the standard deviation of $S_{i}$. $Z_{\frac{a}{2}}$ is obtained from the standard normal distribution function. The null hypothesis is accepted when $\left|Z\right|\leq Z_{\frac{a}{2}}$ and rejected when $\left|Z\right|\geq Z_{\frac{a}{2}}$ (the null hypothesis is no trend of change). In Equation (8), α is the significance level, *** denotes significance at the level of 0.001, ** at the level of 0.01, * at the level of 0.05, and + at the level of 0.01, respectively.

#### 2.6.2. Sen’s Estimator (S-E) Non-Parametric Tests

The S-E non-parametric test is a non-parametric test used to determine how much a variable changed [23]. The S-E test determines the actual magnitude of change in a linear variable in each time series by assuming that a variable’s change is consistent with a linear change.

The $Q_{i}$ of estimating the amplitude of N pairs of data can be calculated by the following formula:

(9) $$Q_{i}=\frac{x_{j}-x_{k}}{j-k}$$

In Equation (9), $x_{j}$ and $x_{k}$ denote the values of *j* and *k* at a given time (j>k). The driving value of the N value of the S-E amplitude change $Q_{i}$ is equal to its median.

If N is an odd number, the magnitude of S-E is calculated as:

(10) $$Q_{med}=\frac{Q_{\left(N+1\right)}}{2}$$

If N is an even number, the magnitude of S-E is calculated by the formula:

(11) $$Q_{med}=\frac{\left[\frac{Q_{N}}{2}+\frac{Q_{\left(N+2\right)}}{2}\right]}{2}$$

$Q_{med}$ is a non-parametric two-tailed test with an alpha confidence interval.

#### 2.6.3. Sequential Version Mann-Kendall (S-M-K) Non-Parametric Test

When doing mutation analysis, the S-M-K test evaluates the relative values of all circumstances in the time series. A time series trend test is typically accompanied by a change-point test ($x_{1},x_{2},\ldots x_{n}$). The null hypothesis $H_{0}$ (observations $x_{i}$ are randomly ordered over time), assumes the absence of any trend, and the alternative hypothesis H1 is relative (the presence of a monotonically increasing or decreasing trend) [24]. $x_{j}(j=1,\ldots ,n)$ and $x_{k}(k=1,\ldots ,j-1)$ are compared in size, and in each set of comparisons, the number of $x_{j}>x_{k}$ is calculated and denoted by $n_{j}$.

The test statistic $t_{j}$ was obtained by the formula:

(12) $$t_{j}=\sum_{1}^{j}n_{j}$$

the test statistics’ mean and variance were:

(13) $$E\left(t\right)=\frac{n\left(n-1\right)}{4}$$

(14) $$Var\left(t_{j}\right)=\frac{\left[j\left(j-1\right)\left(2j+5\right)\right]}{72}$$

the serial values of the statistic $u\left(t\right)$ are calculated by the following equation:

(15) $$u\left(t\right)=\frac{t_{j}-E\left(t\right)}{\sqrt{Var\left(t_{j}\right)}}$$

In Equation (15), $u\left(t\right)$ is the standard variable with zero mean and unit standard deviation. The $u\left(t\right)$ is calculated so that ${UF}_{k}=u(t)$, then ${UB}_{k}=-u(t)$, and if there is an intersection of the curves ${UF}_{k}$ and ${UB}_{k}$ and the intersection is between the critical lines. The moment corresponding to the intersection is the start time of the mutation point.

#### 2.6.4. Hurst Index

The Hurst index can accurately predict future trends in time series. The absolute value approach, aggregated variance method, R/S analysis, periodogram method, Whittle method, residual variance method, and wavelet analysis are some techniques used to estimate the Hurst index. The most popular technique is R/S analysis, a non-parametric analysis technique that does not require assuming that the underlying distribution is Gaussian and that the item being studied has adequate continuity [24].

The principle of R/S analysis is as follows: consider a time series $\left\{x(j)\right\}$ with t=1,2,…. For any positive integer j≥1, define the sequence of polar differences R.

(16) $$R(j)=\underset{1\leq t\leq j}{MaxX(t,j)}-\underset{1\leq t\leq j}{MinX(t,j)}$$

Standard deviation S series:

(17) Sj=1j∑t=1jxt−xj212

if the following connection is true, a dimensionless ratio may be produced by dividing the standard deviation of the observations by the polar deviation (i.e., rescaled polar deviation).

(18) $$\frac{R\left(j\right)}{S\left(j\right)}={\left(aj\right)}^{H}$$

In Equation (18), α is a constant, and there is the Hurst phenomenon in the time series; H is the Hurst index. H takes values in the range (0, 1). When H=0.5, as described above, each climate element is completely independent, and climate change is random. When 0.5<H<1, it indicates that the time series has the characteristics of long-term correlation and that the process is continuous. Suppose that the overall trend of climate factors in the past forecasts an increase in the future and vice versa. And the higher the *H* value, the greater the persistence. When 0<H<0.5, it signifies a long-term relationship between the time series, but the overall tendency in the future is the opposite of the past. In other words, inverse persistence holds when the general increasing trend in the past forecasts the overall decreasing tendency in the future. The strength of the inverse persistence increases as the value of *H* approaches zero [24,25].

#### 2.6.5. Exponential Smoothing Forecasting Method

The exponential smoothing method is widely used for time series forecasting and has shown good forecasting performance in short- and medium-term time series analysis, including the Simple, Holt linear trend, Brown linear trend, and Damped trend models. In the Holt two-parameter smoothing method model, the forecast consists of two parts: the horizontal part, which is based on the horizontal part of the previous period and updated by the simple exponential smoothing method; the other part is the trend part, which is smoothed and adjusted based on the trend part of the previous period and also updated by the simple exponential smoothing method. The two parts are added together to get the forecast for the next period.

The Holt method not only continuously adjusts the horizontal part but also continuously adjusts the trend part, adjusting the forecast in both horizontal and vertical dimensions so it can better respond to changes in the trend. It is calculated by the formula:

(19) $$\begin{array}{c} L_{t+1}=\alpha D_{t}+\left(1-\alpha \right)\left(L_{t}+T_{t}\right) \end{array}$$

(20) $$T_{t+1}=\beta \left(L_{t+1}-L_{t}\right)+\left(1-\beta \right)T_{t}$$

Due to the trend, the Holt method can predict values for multiple periods: the forecast for the nth future period is equal to the horizontal part of the current period plus n times the trend part. The prediction formula is:

(21) $$\begin{array}{c} F_{t+1}=L_{t+1}+T_{t+1} \end{array}$$

where α and β represent the two smoothing parameters affecting the forecasted values; $D_{t}$ represents the actual value; $F_{t+1}$ represents the forecasted value; $L_{t}$ represents the average demand; and $T_{t}$ represents the trend of growth; the former is a smoothing equation for the trend of the time series; the latter is a smoothing equation for the increment of the trend [26].

## 3. Results

### 3.1. Spatial and Temporal Changes and Trends of Water Resources

**Figure 3:**
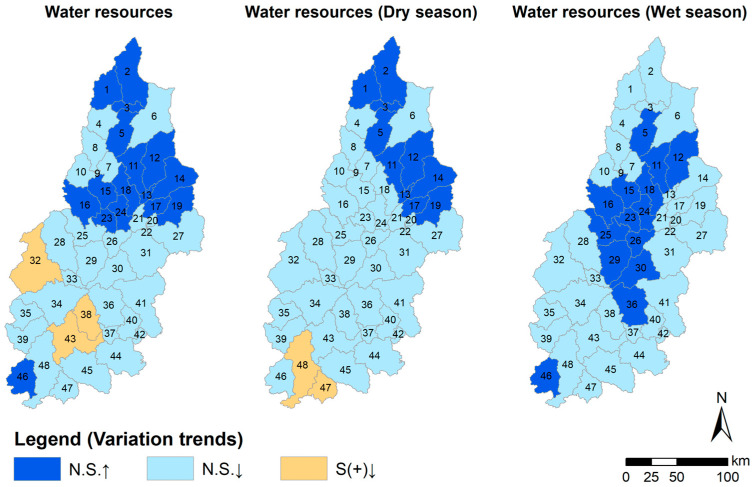
Spatial distribution pattern of variation trend of water resources (10^8^ m^3^) (billion cubic meters) in subbasins from 1970 to 2020. Note a: S indicates significant M-K test; N.S. indicates not significant. ↑ Indicates an increase; ↓ indicates a decrease. Note b: + indicates significant at the α = 0.1 level.

The spatial and temporal dynamic evolution model of water resources in the Hanjiang River Basin was developed by executing the M-K test on the water resources of each of the 48 sub-basins identified by the SWAT model between 1970 and 2020, as shown in Figure 3. Water resource fluctuations are light across the basin, both on a year-round time scale and during the wet and dry seasons, with no significant variation in the situation. Throughout the year, sub-basins with increasing water resource trends are mainly concentrated in the northern region. The rest of the sub-basins show a decreasing trend in water resources.

During the dry season, the sub-basins with an increasing water resource trend decreased relative to the whole year, and most of the sub-basins in the basin had decreasing water resource trend test results. In the wet season, the water resources trend test results in an increase in the distribution of sub-basins that is different from the annual scale and dry season, concentrated in the middle of the basin.

**Figure 4:**
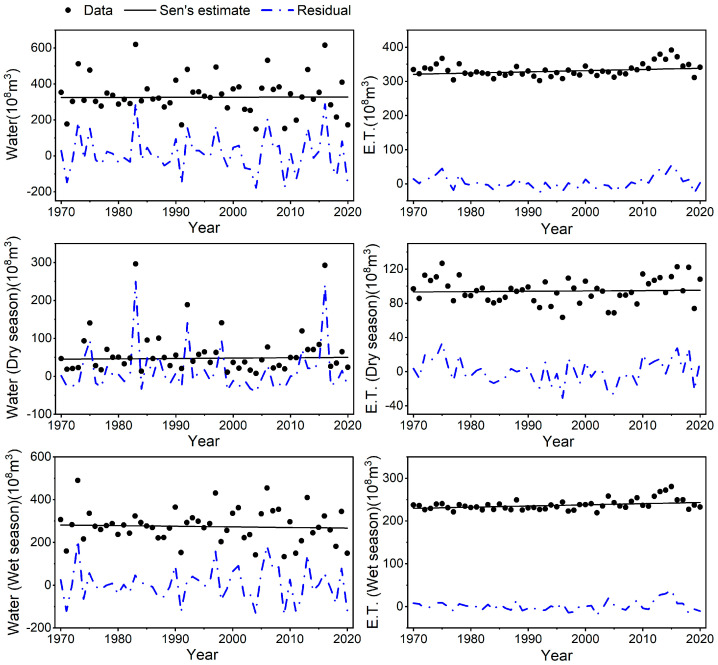
Variation trend of water resources and evapotranspiration from 1970 to 2020.

The water resources in the basin were found to have N.S. results for the entire year, dry season, and rainy season from 1970 to 2020, per the results of the M-K test. This demonstrates that there have been very few changes in the basin’s water supplies over the past 50 years. The S-E test result of 0.06 for the entire year indicates minimal fluctuations in water resources. However, the slope of the fitted straight line (S-E) values for water resources in the dry and wet seasons is similar in magnitude, with opposite positive and negative values (Figure 4, Table 2). The average annual water resources in the basin in the last 50 years were 27.778 billion m^3^ in the wet season and 6.159 billion m^3^ in the dry season (Table 3). The average annual water resources in the wet season were 4.5 times those in the dry season. Assuming that the amount of water resources in the dry season of a year is 10 billion m^3^, the water resources in the wet season are 45 billion m^3^. In the dry season of the second year, water resources will increase by 3.9 billion m^3^, while water resources will decrease by 16.2 billion m^3^ in the wet season. The decrease in water resources in the wet season is greater than that in the dry season. In terms of interannual changes, the water resources in the second year are less than those in the first year. Water resources will tend to decrease.

The S-M-K test indicates that the water supplies in the basin have increased or dropped drastically in a single year over the previous 50 years. Nevertheless, these variations result from abrupt changes in annual precipitation, which are irregular and scattered years of abundance or deficiency. They do not affect the basin’s characteristics, which have not changed considerably for a very long time.

### 3.2. Forecast of Future Trends in Water Resources

The S-E test result for water resources from 1970–2020 in the basin is 0.06, which is positive. However, the Hurst index for water resources is 0.35 < 0.5, which has inverse persistence, indicating that the water resources in the basin tend to change in the opposite direction, which is not significant. The trend is weak and will gradually decline. As a result, the water resources will steadily increase throughout the dry season and change favorably. However, the water resources will slightly decrease during the wet season. The basin’s water resources generally show a pattern of decline, and the trajectory of future changes is not optimistic.

**Figure 5:**
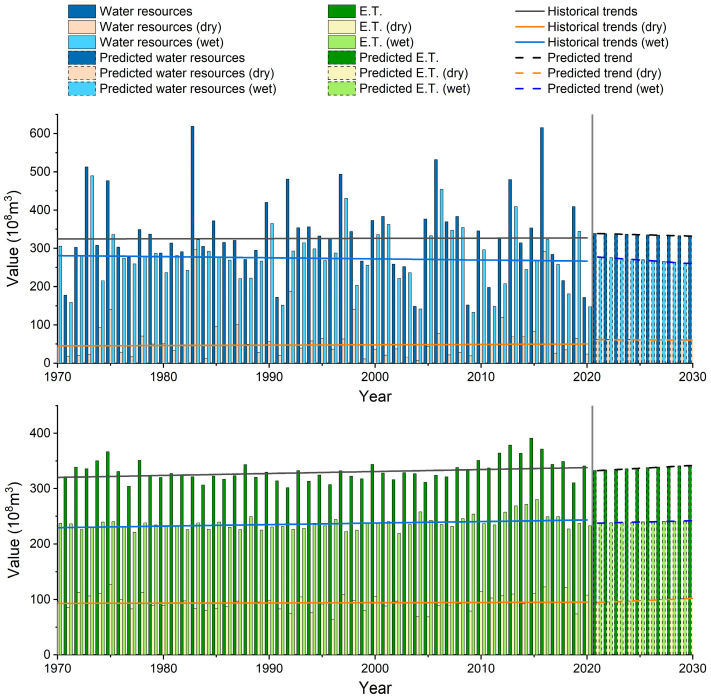
Forecast of water resources and E.T. in the Hanjiang River Basin for the next 10 years.

This result is also verified by the Holt index smoothing prediction. In this study, the trends of water resources and evapotranspiration in the Hanjiang River Basin from 2021 to 2030 are predicted, and the smoothed $R^{2}$ in Table 4 are all high, indicating that the Holt index smoothing model in this study works well. The results indicate that the water resources in the Hanjiang River Basin will change toward a decreasing trend in the next 10 years, with a clear trend of decreasing water resources in the wet season. The change trend of evapotranspiration in the next 10 years is exactly opposite to the change trend of water resources, with a clear upward trend. The analysis of the predicted curve of water resources shows that the water resources fluctuate more in the wet season than in the dry season in the next 10 years (Figure 5).

### 3.3. Driving Mechanism of the Spatial and Temporal Evolution of Water Resources

**Figure 6:**
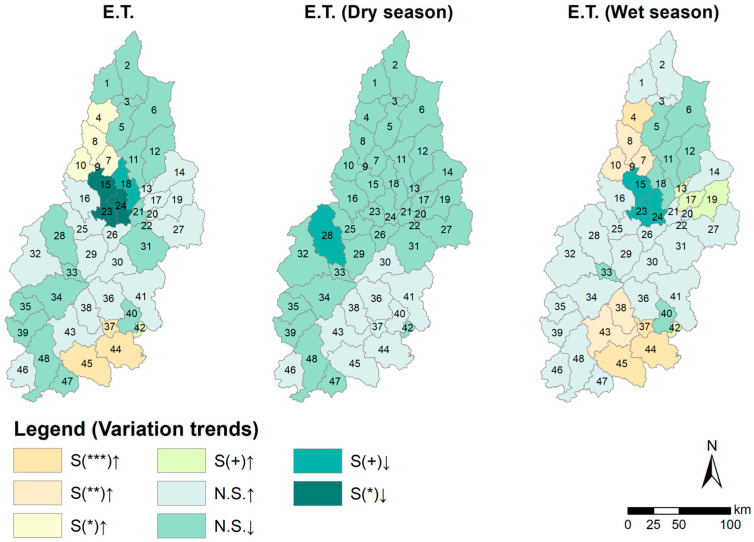
Spatial distribution pattern of variation trend of evapotranspiration in subbasins from 1970 to 2020. Note a: S indicates significant M-K test; N.S. indicates not significant. ↑ Indicates an increase; ↓ indicates a decrease. Note b: *** indicates significant at the α = 0.001 level, ** indicates significant at the α = 0.01 level, * indicates significant at the α = 0.05 level, + indicates significant at the α = 0.1 level.

**Figure 7:**
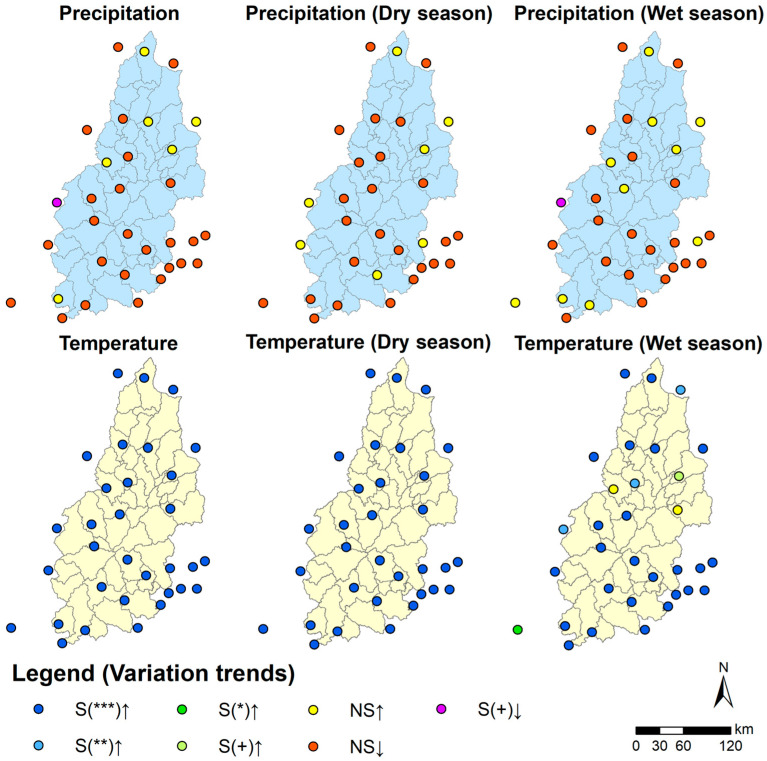
Spatial distribution pattern of variation trend of precipitation and temperature at stations from 1970 to 2020. Note a: S indicates significant M-K test; N.S. indicates not significant. ↑ Indicates an increase; ↓ indicates a decrease. Note b: *** indicates significant at the α = 0.001 level, ** indicates significant at the α = 0.01 level, * indicates significant at the α = 0.05 level, + indicates significant at the α = 0.1 level.

This study’s M-K test and spatial distribution of historical trends were visualized for evapotranspiration (E.T.), precipitation, and temperature in the basin (Figure 6 and Figure 7). The results indicated that E.T. in the basin was significant at the α = 0.01 level on a year-round scale and showed an increasing trend, dry season E.T. was insignificant, and wet season E.T. was significant at the α = 0.01 level and showed an increasing trend. The precipitation change was insignificant throughout the year and in both wet and dry seasons, but the temperature change was significant at α = 0.001 level throughout the year and in both the wet and dry seasons. The range of increases in both temperature and evapotranspiration is significant. The above test results indicate that the decrease in water resources is due to an increase in evapotranspiration (Table 5) caused by the significant increase in air temperature, while the change in precipitation is not significant. The Hurst index indicates that the trend of rising temperatures and rising evapotranspiration is intense, while the change in precipitation is not intense. It means that the trend of rising temperatures, increasing evapotranspiration, and decreasing water in the basin will be maintained, posing a threat to the Basin’s water resources.

In this work, we examined the spatial patterns of temperature and precipitation variations at 32 meteorological stations in the basin from 1970 to 2020 to understand the regional and temporal changes in water resources. Temperatures at the 32 stations in the basin increased significantly at the α = 0.001 level throughout the year and during the dry season. In contrast, during the wet season, the growing trend was spread at the α = 0.001, α = 0.01, α = 0.05, α = 0.1, and N.S. levels, resulting in relatively moderate evapotranspiration and no declining trend of water resources in the area.

A multiple linear regression analysis was performed in this study using evapotranspiration (E.T.), precipitation, air temperature, air pressure, humidity, and wind speed as variables and water resources as dependent variables over 50 years in the Hanjiang River Basin to examine the relationship between these elements and changes in water quantity. Table 6 displays the findings of the analysis. Each element’s VIF value in the table is less than 10, which shows that it agrees with the results of the multivariate linear analysis. The regression fitting equation is $y=548.937+0.355x_{1}-9.483x_{2}-0.540x_{3}-0.478x_{4}-2.342x_{5}+e$ (*e* is the residual). An increase in temperature causes a decrease in water resources, as demonstrated by the unstandardized coefficient B, which indicates that temperature has the greatest influence on the change in water resources and has a strong inhibitory effect. Consistent with the conclusions of the MK test and SE test in the previous section, the main explanation for the decline in water resources in the basin is an increase in evapotranspiration caused by a temperature rise.

## 4. Discussion

### 4.1. Discussion

The 50-year basin condition of the Hanjiang River Basin from 1970 to 2020 is simulated in this study using the SWAT model. Precipitation, temperature, and other climate data are also combined. Finally, statistical tests such as the Mann-Kendall non-parametric test were used to examine the water resources of the basin’s changing trend and anticipate its future trend. In addition to providing a theoretical foundation for managing and planning water resources there, this paper fills a research gap on the Hanjiang River Basin. The study’s results suggest that the basin’s water resources did not grow considerably between 1970 and 2020, and the precipitation exhibited a decreasing trend while temperatures continued to rise. Changes in the basin’s climate increased evapotranspiration, which decreased the average annual surface runoff, base flow, and groundwater recharge, changing the basin’s water resources. Despite climate change and extreme hydrological events, the Hanjiang River Basin’s temperature and evapotranspiration have increased significantly over the past 50 years, yet precipitation and water resources haven’t altered substantially. Evapotranspiration in the wet season of the basin is increasing significantly at the same level of α=0.01 with a trend of an S-E value of 2.76, leading to a decrease in water resources in the wet season and making the basin tend to be dry. The E.T. Hurst index for the wet season is 0.86, indicating that E.T. will continue to increase in the future as well. In the future, water resources will fluctuate little on a year-round scale, but the S-E test result of 1.75 for evapotranspiration and 0.9 for the Hurst index indicate a strong trend of increasing evapotranspiration in the future. If this unfavorable trend continues over time, the Hanjiang River Basin will have less and less water available in the future.

The world experienced an unprecedented number of high-impact climatic extremes between 2001 and 2010, according to the WMO (2013), as a result of the climate system’s unmatched rate of change [27]. The consequences of climate change have been reported to have increased global mean annual average temperatures and altered regional precipitation patterns. It is projected that these effects will continue and worsen in the future. The trend of water resources in the Hanjiang River Basin in this study is consistent with this feature. Changes in precipitation, temperature, and other variables that have an impact on hydrological discharge processes are some of the ways that climate change affects the terrestrial hydrological cycle system [28]. Precipitation, evapotranspiration, runoff, and soil moisture are a few of the components of the water cycle that are severely impacted by climate change; changes in these factors will affect the redistribution of water [11,29].The relationship between precipitation and water resources is positive, while the relationship between temperature and evapotranspiration is negative. Evapotranspiration results from a complex interaction between various environmental factors, including temperature, precipitation, air pressure, humidity, wind speed, and insolation, among which temperature plays a decisive role. The most effective approach to producing runoff and replenishing water resources is through precipitation, which serves as the primary source of runoff recharge. Temperature increases will enhance evapotranspiration while concurrently decreasing the soil’s water content. Global warming accelerates the water cycle, causing rainfall to be more unevenly distributed in time and area and leading to frequent alternations of floods and droughts that affect the uneven spatial and temporal distribution of water resources [27].

However, human activity also affects the hydrological cycle in addition to the natural environment. Land use change significantly affected the basin’s subsurface conditions and changed the functions of soil and water conservation and rainfall collection on the underlying surface, thus affecting the runoff and confluence conditions of the basin. From 1970 to 2020, the forest area in the northern part of the basin increased greatly, which corresponds to the area where the water resources in Section 3.1 increased. The urbanization in the southeastern and central parts of the basin is serious, and the urban land is almost completely transformed from forest and grassland. The forest can conserve water and reduce soil erosion. In addition, forests can convert rainwater into groundwater and increase the total amount of freshwater resources. The reduced wooded area has an impact on evapotranspiration, lateral flow, and vegetation retention mechanisms [30,31], which results in increased runoff [32]. Changes in the sub-base can change soil properties, precipitation retention processes, infiltration processes, surface roughness, and flood frequency [33,34]. The complex underlying surface of the city changes the confluence path of shallow groundwater or subsurface flow, and the complex connection between permeable and impervious areas affects the surface confluence process. Urbanization may also change the shape and structure of the original river network, so that the tributaries gradually disappear [35]. The difference in water resource change trends between the south and north of the Hanjiang River Basin results from this.

This study’s results align with those of earlier studies that created climate change models to investigate future climatic trends and their implications on water resources. They indicate a significant decrease in water supplies in southern China [36]. Miao Lianhua examined the time-series features of runoff in the basin using the Mann-Kendall test and the cumulative distance level method and found that the runoff exhibits a little rising tendency during the dry season while steadily declining during the flood season.

In the face of uneven spatial distribution of water resources, the government should focus on sub-basins with significant declining trends, adopt targeted planning and management, or deploy from water-rich sub-basins. Facing the uneven distribution of water resources in time, the basin authority should seize the point of storing water in the wet season and using water in the dry season, which can effectively prevent drought. Water shortages can be divided into three categories: resource water shortages, water quality water shortages, and engineering water shortages. For the three types of water shortages, the following aspects can be managed. According to the seasonal change characteristics of water resources in the basin and regional demand for water supply, water demand is strictly limited to prevent resource-based water shortages. Strictly prohibit behaviors harmful to the development and use of water resources and strengthen investment in environmental protection in areas with serious water pollution to prevent water quality shortages [37]. Improve the water supply and water transfer network system and build water transfer projects to prevent engineering water shortages. In addition, clear water resources dispatching indicators must be set within water transfer projects, including total water resources control indicators, macro indicators of effective water allocation, and micro indicators of water metering quotas, regular unit water balance tests or water use efficiency assessments, modification of water storage status according to reservoir inflows, climate forecasts, and water storage status at specific time periods, and timely monitoring of water resources use.

### 4.2. Limitations and Prospects

Natural climate change impacts water resources, but so does human activity. More thorough research utilizing land use change data is required to adequately investigate the regional and temporal evolution characteristics of water resources in the Hanjiang River Basin. This study evaluates the overall trend of the water resources in the basin rather than focusing on which component of the water accounts for the largest share of the largest change. On the other hand, the hydrological model’s uncertainty is a crucial factor that determines the efficiency of watershed management.

To further improve the model’s accuracy and address the current study’s limitations, future research is anticipated to incorporate more factors for evaluating the model and feed reservoir data from the basin into the SWAT model. In addition, climate change gradients such as precipitation and temperature are established and input into SWAT for simulation. The ecosystems were delineated to assess the water resources in the Hanjiang River Basin from the perspective of ecosystems and functions and visualize land use and explore the causes of water resource changes in the Hanjiang River Basin in a more refined way.

## 5. Conclusions

This study derives the following conclusions after examining the characteristics and driving mechanisms of water resource trend changes in the Hanjiang River Basin:

(1) Water resources in the basin have fluctuated little over the past 50 years, with dry and wet seasons changing in opposite trends. Evapotranspiration has shown a strong increasing trend in the past and in the future. The forecast results for water resources in the future are reduced.
(2) The changes in water resources in the basin have been unevenly distributed over the last 50 years, with significant spatial changes in the south and smaller changes in other regions of the basin. The difference in water resource change trends in the basin is caused by land use.
(3) The core aspect affecting total water resources in the basin during the past 50 years has been climate change. The main factor contributing to the basin’s diminished water resources is a huge increase in evapotranspiration brought on by a significant rise in temperature. Suppose there isn’t a significant increase in precipitation in the next few years; the sustainability of future water resources in the basin will be gravely jeopardized.

Highly accurate hydrological modeling is an important prerequisite for conducting hydrological research. Grasping the spatial and temporal evolution patterns of water resources in a basin and studying the historical and future trends of water resources are of great importance for scientific management and effective utilization of water resources. The findings of this study can provide scientific references for hydrological modeling and water resource change studies at the global basin scale and provide theoretical support for hydrological response studies of water resources to climate and environmental change impacts.

## Figures and Tables

**Table 1:** Research data and sources.

Data Type	Data Description	Data Source
Digital Elevation Model (DEM) data	90 m resolution	Chinese Academy of Sciences data cloud (http://www.csdb.cn/ (accessed on 1 July 2021))
Land use data	National land use data for 1980, 1990, 2000, 2010, 2020 (30 m resolution)	Land use data were obtained through remote sensing interpretation, and remote sensing data was obtained from the official website of the U.S. Geological Survey (http://earthexplorer.usgs.gov (accessed on 7 July 2021))
Soil data	The soil type of China	Harmony World Soil Database (HWSD) (https://www.fao.org/soils-portal/ (accessed on 10 July 2021))
Meteorological data	Daily-scale data of 32 meteorological stations in Hanjiang River Basin from 1970 to 2020	National Meteorological Science Data Sharing Service Platform (http://data.cma.cn/site/index.html (accessed on 28 June 2021)), Meteorological bureaus of Guangdong, Fujian and Jiangxi
Runoff data	Runoff Data of Chaoan Station from 1980 to 2010	Hanjiang River Basin Administration

**Table 2:** M-K test, S-M-K test, S-E test and Hurst index of each element from 1970 to 2020.

**Element**	**Annual**			**Dry Season**			**Wet Season**			
	**M-K**	**S-M-K**	**S-E**	**M-K**	**S-M-K**	**S-E**	**M-K**	**S-M-K**		**S-E**
Water resources	N.S.	1973 ↑ 2018 ↓ 2020 ↓	0.06	N.S.	1971 ↓ 1974 ↑ 1981 ↓ 2010 ↑ 2017 ↓ 2020 ↓	0.39	N.S.	1972 ↑ 1976 ↓ 1978 ↑ 1985 ↓ 1993 ↑	1998 ↓ 2005 ↑ 2011 ↓ 2013 ↑ 2018 ↓	−0.36
E.T.	S(+) ↑	1980 ↓ 2012 ↑	1.75	N.S.	1971 ↓ 1972 ↑ 1977 ↓ 2015 ↑ 2019 ↓	0.29	S(**) ↑	1987 ↓ 2004 ↑		2.76
**Element**	**Annual**			**Dry Season**			**Wet Season**			
	**Hurst Index**									
Water resources	0.35			0.57			0.47			
E.T.	0.90			0.79			0.86			
Precipitation	0.36			0.58			0.48			
Temperature	0.95			0.88			0.89			
Air pressure	0.95			0.95			0.93			
Humidity	0.99			0.95			0.99			
Wind speed	0.98			0.96			1.00			

Note a: S indicates significant M-K test, N.S. indicates not significant. ↑ Indicates an increase, ↓ indicates a decrease. Note b: ** indicates significant at the α = 0.01 level, + indicates significant at the α = 0.1 level.

**Table 3:** Average water resources and E.T. (10^8^ m^3^) in the Hanjiang River Basin from 1970 to 2020.

	Annual	Dry Season	Wet Season
Average water resources	338.86	61.59	277.78
Average E.T.	332.67	94.88	237.79

**Table 4:** Stable R^2^ results of Holt exponential smoothing.

	Water Resources	Water Resources (Dry Season)	Water Resources (Wet Season)	E.T.	E.T. (Dry Season)	E.T. (Wet Season)
Stable R^2^	0.849	0.840	0.846	0.737	0.830	0.672

**Table 5:** M-K, S-M-K, and S-E test results of climatic factors from 1970 to 2020.

Element	Annual			Dry Season			Wet Season		
	M-K	S-M-K	S-E	M-K	S-M-K	S-E	M-K	S-M-K	S-E
Precipitation	N.S.	1973 ↑ 1976 ↓ 2015 ↑ 2017 ↓	−0.54	N.S.	1974 ↑ 1976 ↓ 1983 ↑ 2007 ↓ 2015 ↑ 2017 ↓	0.10	N.S.	1972 ↑ 1977 ↓ 1985 ↓ 2006 ↑ 2013 ↑ 2019 ↓	−0.26
Temperature	S(***) ↑	/	5.15	S(***) ↑	/	4.55	S(***) ↑	/	3.93
Air pressure	S(***) ↓	/	−0.61	S(***) ↓	/	−0.65	S(***) ↓	/	−0.58
Humidity	S(***) ↓	/	−0.07	S(*) ↓	/	−0.07	S(***) ↓	/	−0.07
Wind speed	S(**) ↓	/	−0.08	S(***) ↓	/	−0.09	S(**) ↓	/	−0.06

Note a: S indicates significant M-K test; N.S. indicates not significant. ↑ Indicates an increase; ↓ indicates a decrease. Note b: *** indicates significant at the α = 0.001 level, ** indicates significant at the α = 0.01 level, * Indicates significant at the α = 0.05 level.

**Table 6:** Multiple regression calculation results of water resources and climate factors.

	Unstandardized Coefficients		95.0 % Confidence Intervals for B		Correlation			Collinearity Statistics	
	B	Std. Error	Upper Limits	Lower Limits	Zero-Order	Partial	Part	Tolerance	VIF
Water resources (*y*)	548.937	3940.00	−7386.63	8484.51					
Precipitation (*x*_1_)	0.355	0.01	0.33	0.38	0.98	0.97	0.913	0.88	1.14
Temperature (*x*_2_)	−9.483	8.49	−26.58	7.62	−0.12	−0.16	−0.036	0.61	1.63
Air pressure (*x*_3_)	−0.540	3.91	−8.42	7.34	0.13	−0.02	−0.004	0.52	1.94
Humidity (*x*_4_)	−0.478	1.92	−4.35	3.40	0.33	−0.04	−0.008	0.70	1.43
Wind speed (*x*_5_)	−2.342	1.99	−6.35	1.67	0.01	−0.17	−0.038	0.80	1.25

## Data Availability

Digital elevation Model data can be accessed at the Chinese Academy of Sciences data cloud (http://www.csdb.cn/ (accessed on 1 July 2021)). Land use data can be accessed at the U.S. Geological Survey (http://earthexplorer.usgs.gov (accessed on 7 July 2021)). Soil data can be accessed at the Harmony World Soil Database (https://www.fao.org/soils-portal/ (accessed on 10 July, 2021)). Meteorological data can be accessed at the National meteorological science data sharing service platform (http://data.cma.cn/site/index.html (accessed on 28 June 2021)). All the data presented in this study are available on request from the corresponding author.
